# 

**Published:** 1895-09

**Authors:** 


### TABLE OF CONTENTS.

ORIGINAL COMMUNICATIONS. PAGE

Which Method of Root-Canal Filling will completely obliterate Space? By Safford G. Perry, D.D.S., New York . . . . . . . . . . . . . . . . 527

Syphilis, with Special Reference to the Relations of the Dental Profession to the Disease. By Henry A. Pulsford, M.D. . . . . . . . . . . . . 536

Reply to Dr. W. H. Trueman. By Dr. I. Norman Broomell, Philadelphia 543

A New Method of attaching Artificial Crowns to Badly Decayed Roots. By George H. Chance, D.D.S., Portland, Oregon . . . . . . . . . . 545

A Plea for Moderation. By Dr. B. Holly Smith, Baltimore, Md. . . . 547

The Census of 1890 and the Dentists. By Dr. William Shaw Twilley, Baltimore, Md. . . . . . . . . . . . . . . . . . . . . . . . . . . . 551

A National Dental Museum. By Dr. H. B. Noble, Washington, D.C. . . 555

REPORTS OF SOCIETY MEETINGS.

New York Odontological Society . . . . . . . . . . . . . . . . . . . 556

Odontological Society of Pennsylvania . . . . . . . . . . . . . . . . . 577

EDITORIAL.

Dental Training . . . . . . . . . . . . . . . . . . . . . . . . . . . 586

OBITUARY.

Augustus Woodruff Brown, D.D.S. . . . . . . . . . . . . . . . . . . 588

NOTES AND COMMENTS . . . . . . . . . . . . . . . . . . . . . . . . 589

